# Loss of IRF2BP2 in Microglia Increases Inflammation and Functional Deficits after Focal Ischemic Brain Injury

**DOI:** 10.3389/fncel.2017.00201

**Published:** 2017-07-19

**Authors:** Shelly A. Cruz, Aswin Hari, Zhaohong Qin, Pascal Couture, Hua Huang, Diane C. Lagace, Alexandre F. R. Stewart, Hsiao-Huei Chen

**Affiliations:** ^1^Ottawa Hospital Research Institute Ottawa, ON, Canada; ^2^Brain and Mind Institute, University of Ottawa Ottawa, ON, Canada; ^3^Canadian Partnership for Stroke Recovery Ottawa, ON, Canada; ^4^Cellular and Molecular Medicine, University of Ottawa Ottawa, ON, Canada; ^5^University of Ottawa Heart Institute Ottawa, ON, Canada; ^6^Biochemistry, Microbiology and Immunology, University of Ottawa Ottawa, ON, Canada; ^7^Medicine, University of Ottawa Ottawa, ON, Canada

**Keywords:** ischemic brain injury, inflammation, microglia, interferon beta, sensory-motor function, photothrombosis

## Abstract

Ischemic stroke causes neuronal cell death and triggers a cascade of inflammatory signals that contribute to secondary brain damage. Microglia, the brain-resident macrophages that remove dead neurons, play a critical role in the brain’s response to ischemic injury. Our previous studies showed that IRF2 binding protein 2 (IRF2BP2) regulates peripheral macrophage polarization, limits their inflammatory response and reduces susceptibility to atherosclerosis. Here, we show that loss of IRF2BP2 in microglia leads to increased inflammatory cytokine expression in response to lipopolysaccharide challenge and impaired activation of anti-inflammatory markers in response to interleukin-4 (IL4) stimulation. Focal ischemic brain injury of the sensorimotor cortex induced by photothrombosis caused more severe functional deficits in mice with IRF2BP2 ablated in macrophages/microglia, associated with elevated expression of inflammatory cytokines in the brain. These mutant mice had larger infarctions 4 days after stroke associated with fewer anti-inflammatory M2 microglia/macrophages recruited to the peri-infarct area, suggesting an impaired clearance of injured tissues. Since IRF2BP2 modulates interferon signaling, and interferon beta (IFNβ) has been reported to be anti-inflammatory and reduce ischemic brain injury, we asked whether loss of IRF2BP2 in macrophages/microglia would affect the response to IFNβ in our stroke model. IFNβ suppressed inflammatory cytokine production of macrophages and reduced infarct volumes at 4 days after photothrombosis in wild type mice. The anti-inflammatory effect of IFNβ was lost in IRF2BP2-deficient macrophages and IFNβ failed to protect mice lacking IRF2BP2 in macrophages/microglia from ischemic injury. In summary, IRF2BP2 expression in macrophages/microglia is important to limit inflammation and stroke injury, in part by mediating the beneficial effect of IFNβ.

## Introduction

Ischemic stroke occurs from an abrupt deprivation of oxygen and nutrients to the brain. The ensuing neuronal cell death triggers a cascade of inflammatory responses contributing to secondary brain damage. Cerebral ischemia engages both innate and adaptive immunity. The early innate immune response relies on microglia, perivascular macrophages, blood monocytes, mast cells, neutrophils and dendritic cells, whereas adaptive immunity relies on high-affinity receptors and antigen-driven clonal cell expansion that needs 7–10 days to develop and does not impact on the acute phase of damage (Iadecola and Anrather, 2011).

The interaction between neurons and microglia, the brain-resident macrophages, plays a critical role in how the brain responds to ischemic injury. Microglia and macrophages are derived from the myeloid lineage (Hess et al., 2004; Ginhoux et al., 2013). Both macrophages and microglia are activated by focal ischemic brain injury. Activated microglia surrounding the infarct area engulf and remove dead neurons. At the same time, they secrete inflammatory cytokines and chemokines to recruit more circulating immune cells to the injured site, including peripheral monocytes and granular cells, after disruption of the blood-brain barrier.

Microglia and macrophages share a common lineage, but unlike macrophages residing in peripheral tissues that are replenished from bone marrow, microglia proliferate from the original progenitors that are trapped in the brain after the blood-brain barrier forms (Ajami et al., 2007; Mildner et al., 2007). Interestingly, a recent study using direct RNA sequencing showed that despite extensive overlap in the basal gene expression profiles of microglia and macrophages, a number of genes are differentially expressed between these two cell types (Hickman et al., 2013), suggesting that segregation across the blood brain barrier modifies their gene expression program.

Ischemic brain injury activates the pro-inflammatory transcription factor IRF1 (interferon regulatory factor 1). IRF1 expression is markedly elevated in both microglia and neurons of mice and humans after stroke, peaking on day 4 after ischemic brain injury (Iadecola et al., 1999; Alexander et al., 2003). IRF1 activates many inflammatory cytokines and promotes M1 macrophage polarization (Xie et al., 2016). At the same time, IRF1 function is counteracted by IRF2, a related transcription factor that binds to the same DNA sequences as IRF1 but acts as a transcriptional repressor (Zhang et al., 2002). The transcriptional suppression effect of IRF2 is mediated by its interaction with co-repressors including IRF2 binding protein 2 (IRF2BP2), IRF2BP1 and IRF2BPL (Childs and Goodbourn, 2003; Heger et al., 2007).

We recently identified a novel function of IRF2BP2 as a key regulator of macrophage polarization: it promotes activation of anti-inflammatory (M2) marker genes and inhibits pro-inflammatory (M1) markers (Chen et al., 2015). Bone Marrow-derived Macrophages (BMDMs) isolated from LysMCre/IRF2BP2flox mice do not express IRF2BP2 and have impaired cholesterol handling due to their inflammatory phenotype. IRF2BP2-deficient macrophages have a heightened inflammatory response when challenged with lipopolysaccharides (LPS), whereas their response to interleukin-4 (IL4)-induced activation of anti-inflammatory M2 genes is impaired. Our studies further demonstrated that ablation of IRF2BP2 in macrophages worsens atherosclerosis in mice, and a deletion variant that lowers IRF2BP2 expression predisposes to coronary artery disease in humans (Chen et al., 2015). Given known differences in basal gene expression between macrophages and microglia (Hickman et al., 2013), it was important to determine whether IRF2BP2 controls microglia polarization in a similar fashion as in macrophages.

Preclinical stroke studies show that increased pro-inflammatory M1 polarization is associated with a larger infarct and worse stroke outcome whereas anti-inflammatory M2 polarization resolves inflammation, limits stroke injury progression and promotes tissue regeneration and recovery from stroke injury (Frieler et al., 2011; Iadecola and Anrather, 2011). Thus, identifying molecular mechanisms that promote M2 polarization to resolve inflammation and enhance tissue repair may provide therapeutic targets to limit stroke injury and improve stroke recovery.

Interferon beta (IFNβ) was reported to attenuate inflammatory activation and limit infarction after ischemic brain injury (Inacio et al., 2015; Kuo et al., 2016). However, others have failed to see this effect (Maier et al., 2006) and the therapeutic utility of IFNβ on stroke recovery remains uncertain. IFNβ inhibits inflammatory cytokine expression in co-cultures of microglia and astroglia (Hinkerohe et al., 2005), in part through IRF2 activation (Buttmann et al., 2007). Studies in the liver have shown that elevated IRF1 expression worsens hepatic ischemic injury (Ueki et al., 2010), whereas elevated expression of IRF2 is protective (Klune et al., 2012). Here, we tested whether disruption of the repressor function of IRF2 by selectively ablating its corepressor IRF2BP2 in microglia and macrophages would have a deleterious effect on ischemic brain injury. We also tested whether IFNβ would limit stroke injury and whether IRF2BP2 is required for the effect of IFNβ.

## Materials and Methods

### Animals

LysMCre/IRF2BP2flox mice that ablate IRF2BP2 in the myeloid lineage (Chen et al., 2015) were bred into the C57BL6 background, fed with regular chow, and randomly assigned to experimental groups. The animal care and use committee of the University of Ottawa approved all procedures performed with these mice.

### Photothrombotic Stroke Surgery and IFNβ Treatment

The photothrombotic focal stroke method was performed as described by Watson et al. (1985). In brief, 2 month-old male mice were anesthetized in an induction chamber ventilated with 5% isoflurane gas, and then transferred to a stereotaxic frame and maintained at 1.5% isoflurane using a nose mask. A midline incision of the scalp was made to expose a 1 × 1 mm region of the skull. Five minutes after administration of Rose Bengal (10 mg/ml in PBS, made fresh by vortexing and filtered, 100 mg/kg, i.p.), a collimated green laser (532 nm wavelength at 20 mW, Beta Instruments) was placed 2 cm above the skull, positioned 0.7 mm anteroposterior and 2 mm mediolateral relative to the Bregma and used to illuminate the skull for 10 min, to initiate photothrombosis. Cyanoacrylate glue was used to close the wound, and mice were monitored until they regained consciousness. For IFNβ treatment, 30 min after photothrombosis, mouse recombinant IFNβ (10,000 units, Sigma) was delivered by intravenous injection via the tail vein in 100 μl saline.

### Quantification of Infarction

Infarct volumes were determined by cresyl violet staining to reveal Nissl bodies of living neurons in 10 coronal sections (20 μm thick) sampled every 10 sections over a 2 mm distance overlapping the area of infarction, as described previously (Schock et al., 2008). Infarct volume was calculated by stacking infarct areas in serial sections using the ImageJ software (NIH). Lesion volumes were normalized to brain volumes of the corresponding sections to control for edema. Investigators were blinded to genotype.

### Primary Neonatal Microglia Isolation and Culture

Microglia from IRF2BP2KO and littermate control mouse pups were cultured as described (Valdearcos et al., 2014). Briefly, cortices from postnatal mice (day 1–3) were stripped of meninges, minced into small pieces, and vortexed in DMEM (Wisent) to kill neurons before being sieved through a 70 μm strainer. Cell suspensions from each pup were seeded separately onto poly-D-Lysine (Sigma) coated T-75 tissue culture flasks (Falcon) in complete DMEM medium with 10% FBS (Wisent). Culture medium was gently replaced every 2 days until the microglia were visible on the astrocyte monolayer (~7–10 days). Loosely attached microglia were then shaken off at 250 rpm for 30 min and cultured in DMEM supplemented with 10% FBS for 3 h. After microglia were attached, medium was changed to Neurobasal (GIBCO) with N1 supplement (Sigma) and cells were then ready for further treatment with LPS (100 ng/ml, Sigma) or IL4 (20 ng/ml, Sigma) for 48 h or for the phagocytosis assay (see below). The purity of these cells upon plating was in excess of 95% as confirmed by CD11b staining via FACS (data not shown).

### Microglia Phagocytosis Assay

Primary cultured microglia (8 × 104 cells/well; 24 well plate with coverslips) from IRF2BP2KO and littermate control mice were incubated with Flash-Red conjugated microspheres (Bangs Laboratories, Inc.) in the presence or absence of 100 ng/ml LPS for 24 h. Cells were washed three times with cold 1× PBS, fixed with 4% paraformaldehyde and washed several times before incubated with Iba1 antibody (#019-19741, Wako), followed by Cy2-conjugated secondary antibody (#711-225-152, Jackson ImmunoResearch). Internalization of these microspheres was visualized on a Zeiss Z1 AxioImager fluorescence microscope. The number of microspheres per cell was counted as an index of phagocytic activity. Investigators were blinded to genotype.

### *Ex Vivo* Adult Microglia Isolation for FACS

Isolation of microglia after stroke was done as described previously, with modifications (Ren et al., 2011). Following mechanical dissociation of the brain, a single cell suspension was obtained by chopping the tissue with tweezers and squeezing the tissue through a 20 gauge needle. The suspension with cells was centrifuged at 1500 *g* for 5 min and passed through a 70 um filter. The cells were then layered on top of 30% percoll (Sigma) and 70% percoll. The gradient was centrifuged at 800 *g* for 40 min at room temperature without brake. The cells at the 70%–30% percoll interface was collected and washed in culture media for 10 min at 1500 rpm at 4°C. The cells were re-suspended in PBS and stained with the following antibodies in the presence of CD16/32 blocking antibody (BD, 553142). Samples were read on the BD Fortessa or Moflo Astrios.

### FACS Antibodies and Gating

The gating for microglia was done in the following manner to separate resident and incoming cell populations. Resident microglia: CD45^int^Ly6c^−^780 Viability dye^−^, CD11b^lo/+^; migratory cells: CD45^+^Ly6c^+^ 780 Viability dye^−^, CD11b^lo/+^. The number of cells expressing MHCII and CD206 (from MHCII negative cells) were calculated using FlowJo, Treestar Inc. Antibodies for FACS analysis were as follows: MHCII-PECy7 (25-5321-82), CD11b-PerCP-cy5.5 (45-0112-82), Ly6c-APC (17-5932-82), CD45-eflour 450 (48-0451-82) all purchased from eBioscience and CD206-PE (141706) from Biolegend. Fixable Viability Dye eFluor^®^ 780 was also from eBioscience.

### Bone Marrow-Derived Macrophage (BMDM) Culture and IFNβ Treatment

BMDM were cultured as we described (Chen et al., 2015) and treated with IFNβ (100 unit/ml) for 2 h prior to stimulation with LPS (10 ng/ml, Sigma) for 4 h. RNA was isolated for RT-qPCR analysis.

### Quantitative Polymerase Chain Reaction

Total RNA from microglia, BMDM or brain tissue was extracted using the Qiagen RNeasy Mini Kit. Reverse transcription-quantitative polymerase chain reaction (RT-qPCR) was conducted as described previously (Pandey et al., 2013), and the results were normalized to GAPDH or actin. Primers used for qPCR: Ccl2: (F) 5′-CACTCACCTGCTGCTACTCATTC-3′, (R) 5′-TCTTTGGGACACCTGCTG-3′. iNos: (F) 5′-AGCCCT CACCTACTTCCTG-3′, (R) 5′-TCTCTGCCTATCCGTCTC-3′, TNFα: (F) 5′-CCACCACGCTCTTCTGTCTAC-3′, (R) 5′-AGGGTCTGGGCCATAGAACT-3′. IL1β: (F) 5′-CAGGCTC CGAGATGAACAA-3′, (R) 5′-CCCAAGGCCACAGGTATTT-3′. Arg1: (F) 5′-TCACCTGAGCTTTGATGTCG-3′, (R) 5′-CTG AAAGGAGCCCTGTCTTG-3′. CD206: (F) 5′-CAAGGAAGGT TGGCATTTGT-3′, (R) 5′-CCTTTCAGTCCTTTGCAAGC-3′. YM1: (F) 5′-GGGCATACCTTTATCCTGAG, (R) 5′-CCACT GAAGTCATCCATGTC-3′. GAPDH: (F) 5′-TGTTCCTACCC CCAATGTGT-3′, (R) 5′-TGTGAGGGAGATGCTCAGTG-3′. actin: (F) 5′-CCTTCTGACCCATTCCCACC, reverse 5′-GCTTCTTTGCAGCTCCTTCG-3′.

### Antibodies for Immunoblot and Immunofluorescence

Protein extraction and Western blot analysis were performed as described (Schock et al., 2008). A custom rabbit antibody against IRF2BP2 was described previously (Teng et al., 2011). Cryostat sections (20 μm) were subjected to cresyl violet staining and immunofluorescence. Immunofluorescence images were acquired on a Zeiss Z1 fluorescent microscope. Primary antibodies used and their dilutions are: MHCII (Biolegend #107602, anti-rat 500× dilution), CD68 (Santa Cruz, sc70761, anti-mouse, 500×), CD206 (Santa Cruz, sc34577, anti-goat, 500×), Iba1 (WAKO, #019-19741, anti-rat, 500×), NeuN (Millipore MAB377, anti-mouse, 500×), and GFAP (Santa Cruz, sc170, anti-goat, 500×). Cy2-, cy3-, cy5-conjugated secondary antibodies (Jackson Labs) were used at 1000× dilution. For immunofluorescence images, three independent fields at 20× magnification from six sections were imaged and CD206+ and CD68+ cells counted using imageJ.

### Adhesive Removal Test

Sensory-motor function was measured by removal of adhesive tape placed on the palms of mice as described online-only supplement, as described (Bouet et al., 2009). Mice in their home cage were placed in a testing room for about 30 min to allow habituation. Before testing, the mice were transferred to a new clean cage and the home cage was used as the testing cage. For testing, an adhesive tape cut into squares enough to cover the hairless part of the paw was placed and pressed gently into the left and right forelimb paw of a restrained mouse. Then mouse was placed in the testing cage and timed immediately by stopwatch with a maximum of 2 min per trial period. Data collected includes left and right contact time and left and right removal time. Mice were trained 6 days before stroke and testing was done 4 days post-stroke. Investigators were blinded to genotype.

### Statistical Analysis

All results are presented as means ± SEM. For between-group comparisons of fold changes, values were normalized by log transformation whereas percentages were normalized by arcsin transformation and a two-tailed Student’s *t* test was applied. Differences in means were considered significant at *p* < 0.05.

## Results

### Skewed Polarization of IRF2BP2-Deficient Microglia Towards an Inflammatory M1 Phenotype

**Figure 1:**
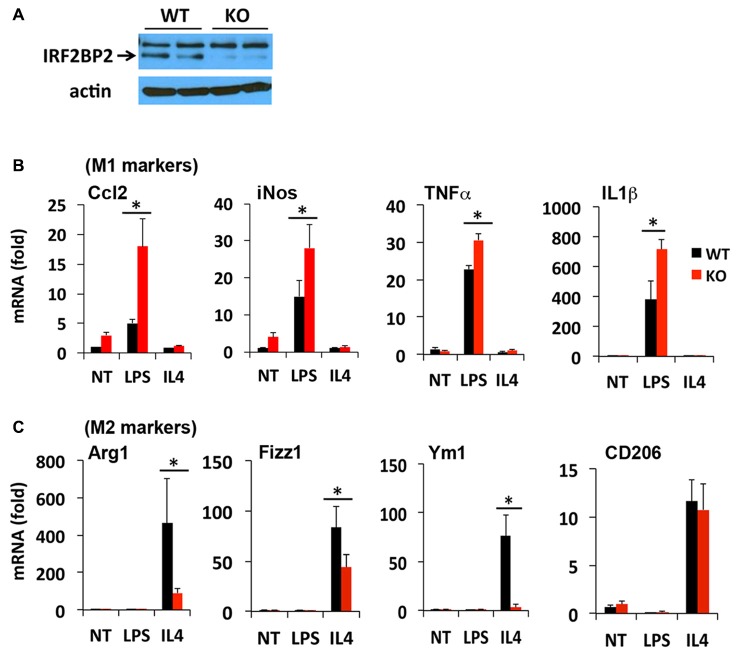
Ablation of IRF2 binding protein 2 (IRF2BP2) skews microglia activation towards an inflammatory phenotype. **(A)** Immunoblot shows loss of IRF2BP2 protein in microglia from LysMCre/IRF2BP2flox (KO) mice. WT, littermate controls. IRF2BP2-deficient microglia have markedly increased expression of (M1) inflammatory markers inducible nitric oxide synthase (iNOS), Ccl2, and the cytokines Tumor Necrosis Factor-α (TNFα) and IL1β after lipopolysaccharide (LPS) challenge **(B)** but a reduced response of anti-inflammatory (M2) markers Arginase 1 (Arg1), Fizz1, Ym1 to interleukin-4 (IL4) stimulation **(C)**. NT, non-treated. *N* = 3 mice/group. *p < 0.05.

The LysMCre transgene expresses Cre recombinase under the control of the endogenous LysM promoter in macrophages (Ye et al., 2003) and microglia (Frieler et al., 2011; Hamner et al., 2015). We reported previously that LysMCre/IRF2BP2flox (IRF2BP2KO) mice ablate IRF2BP2 in their macrophages (Chen et al., 2015). To confirm that IRF2BP2 expression is also ablated in brain microglia, we cultured primary microglia isolated from IRF2BP2KO and littermate control (WT) neonatal mice. Immunoblot analysis revealed that IRF2BP2 protein expression was ablated in microglia of KO mice (Figure 1A). Trace residual IRF2BP2 protein likely reflects the few non-microglia cells that contaminate these primary cultures.

We next compared how loss of IRF2BP2 affects microglia polarization in response to LPS and IL4 stimuli. Both microglia and macrophages can become polarized to a pro-inflammatory M1 (induced by interferon gamma or LPS) or an anti-inflammatory M2 subtype (induced by IL4). M1 markers include inducible nitric oxide synthase (iNOS) and the pro-inflammatory cytokines Ccl2, Tumor Necrosis Factor-α (TNF-α) and IL1β and cell surface marker MHCII, whereas M2 markers include Arginase 1 (Arg1), CD206 (aka Mrc1, Mannose receptor, C type 1), YM1 and Fizz1 (Raes et al., 2002; Cherry et al., 2014; Okuneva et al., 2015).

In contrast to what we observed previously in BMDMs, primary microglia from KO mice did not express higher levels of the inflammatory cytokines TNFα and IL1β at baseline (NT, non-treated; Figure 1B), although this may reflect differences in culture conditions for macrophages and microglia. However, in response to LPS stimulation, a much more robust M1 activation of iNOS, Ccl2, TNFα and IL1β was detected in IRF2BP2KO microglia (Figure 1B). On the other hand, IL4-induced activation of the alternative (anti-inflammatory, M2 subtype) markers Arg1, YM1 and Fizz1 was impaired in KO microglia (Figure 1C). Interestingly, IL4 induces robust expression of CD206 in the IRF2BP2-deficient microglia (Figure 1C), unlike in IRF2BP2-deficient macrophages (Chen et al., 2015).

### Delayed Regression of Infarct Lesion in IRF2BP2 Mice after Photothrombosis- Induced Focal Ischemic Injury

**Figure 2:**
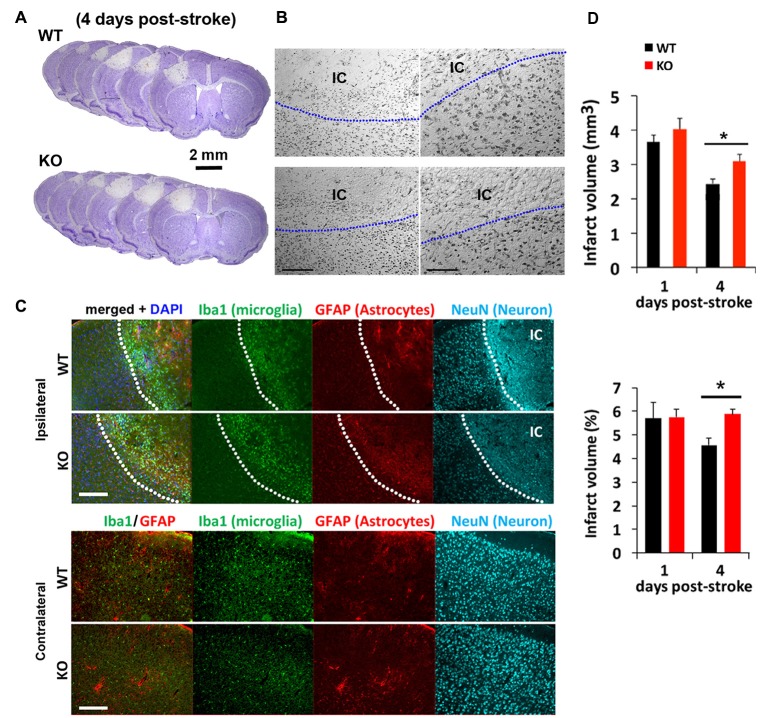
Photothrombosis induced focal ischemic injury to the sensorimotor cortex persists in IRF2BP2KO mice at 4 days. **(A)** Cresyl violet staining reveals lesion area where neurons are lost and devoid of stain. Representative brain sections from IRF2BP2KO (KO) and littermate control mice (WT) 4 days after photothrombosis-induced stroke injury. **(B)** High power magnification showing the clear demarcation of the ischemic core (IC). Left scale bar, 200 μm; right scale bar, 100 μm. **(C)** Immunofluorescence labeling of Iba+ microglia recruited to the IC and activation of astrocytes revealed by glial fibrillary acidic protein (GFAP). Neurons (NeuN+) are absent in the IC. Scale bar, 200 μm. The corresponding region of the contralateral (non-ischemic) side, with few Iba1+ microglia, is shown for comparison. **(D)** Lesion regression is delayed in KO mice. *N* = 10–15 mice/group. *p < 0.05.

We next tested how mice lacking IRF2BP2 in microglia would respond to ischemic brain injury to the left sensorimotor cortex induced by photothrombosis (Figure 2). Cresyl violet staining revealed a similar-sized infarction lesion in WT and KO mice 1 day after stroke (Figures 2A,B). Immunofluorescence staining at 4 days showed a clear demarcation of NeuN+ neurons, absent within the ischemic core (IC; Figure 2C). By 4 days, lesion volumes regressed by 25% in littermate control mice, but failed to regress in KO mice (Figure 2D). Thus, IRF2BP2 ablation in microglia/macrophages appears to delay tissue repair.

### IRF2BP2 Is Required for Microglia/Macrophage Recruitment to the Peri-Infarct Area

**Figure 3:**
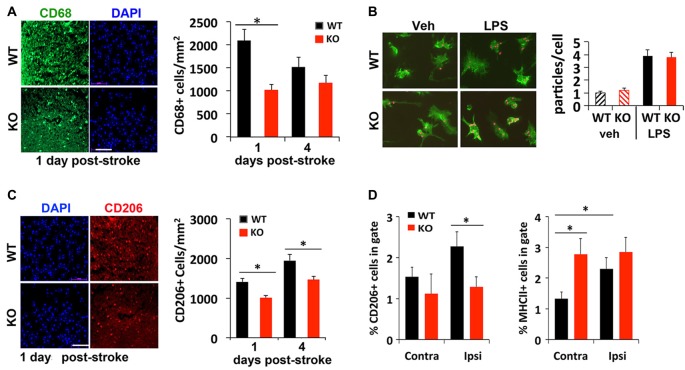
Reduced recruitment of M2 microglia/macrophages in IRF2BP2KO mice after stroke. **(A)** Fewer CD68+ cells, a marker for microglia/macrophages, in the peri-infarct area of IRF2BP2KO mice compared to their littermate controls (WT). *N* = 6 mice/group. **(B)** Microglia from IRF2BP2KO mice engulf similar numbers of red microparticles as littermate controls, with or without LPS stimulation (100 ng/ml, 24 h). *N* = 3 mice/group. **(C)** Fewer anti-inflammatory M2 (CD206+) microglia in the peri-infarct area of KO mice. *N* = 6 mice/group. Scale bar, 100 μm. **(D)** FACS analysis of CD206+ (M2) and MHCII+ (M1) microglia isolated from contralateral and ipsilateral cortex 4 days after stroke. Sham, sham operated mice. *N* = 4 mice/group. *p < 0.05.

Injured or dead neurons activate local microglia that secrete chemokines and cytokines to recruit more immune cells to help clear the dead cell debris for tissue repair. To compare whether a deficit in this process may contribute to delayed regression of lesion 4 days after stroke, immunostaining was used to examine different subtypes of microglia recruited to the peri-infarct area after stroke. We found 50% fewer CD68+ cells, a marker for microglia/macrophages, present in the peri-infarct area of KO mice compared to littermate control mice 1 day after photothrombosis (Figure 3A). An *in vitro* phagocytosis assay showed that KO microglia engulf fluorescent-labeled microparticles just as efficiently as WT microglia (Figure 3B), suggesting that the early deficit in phagocytic cell numbers rather than their ability to phagocytose may account for delayed infarct repair.

Immunostaining showed that significant numbers of CD206+ M2 microglia are recruited to the peri-infarct area 1 day after stroke. However, fewer CD206 cells were detected in KO mice at 1 and 4 days post-stroke, compared to littermate controls (Figure 3C). FACS analysis of microglia isolated 4 days after stroke confirmed fewer CD206+ cells on the ipsilateral (stroke) side in KO compared to WT mice (Figure 3D). Moreover, FACS analysis revealed a higher percentage of M1 (MHCII+) microglia on the stroke side (ipsilateral) compared to the contralateral side in WT mice. Interestingly, KO mice showed a similar high percentage of MHCII+ inflammatory microglia in both hemispheres after stroke (Figure 3D).

### Stroke Markedly Induces Inflammatory Cytokines in IRF2BP2KO Mice

**Figure 4:**
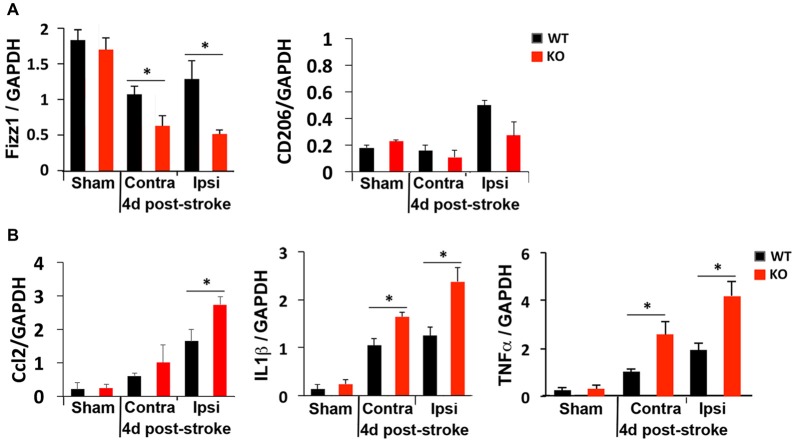
Global elevation of inflammatory cytokines after photothrombosis is worse in KO mice. RT-qPCR revealed **(A)** reduced levels of the anti-inflammatory gene Fizz1 and **(B)** elevated levels of the inflammatory cytokines Ccl2, IL1β and TNFα in both contralateral (Contra) and ipsilateral (Ipsi) sides of KO cortex after ischemic injury. *N* = 4–5 mice/group. *p < 0.05.

We next compared M1 and M2 gene expression 4 days after photothrombosis in the cortical hemisphere ipsilateral to the stroke vs. the contralateral side of the cortex by qRT-PCR. Consistent with immunostaining and FACS analysis that showed fewer anti-inflammatory M2 microglia but more inflammatory M1 microglia at the peri-infarct area of KO mice (Figure 3), lower expression of an M2 gene (Fizz1) and higher levels of M1 genes (Ccl2, TNFα, IL1β) were detected in the ipsilateral cortex of KO mice (Figure 4).

### Absence of IRF2BP2 in Microglia Worsens Stroke Outcome

**Figure 5:**
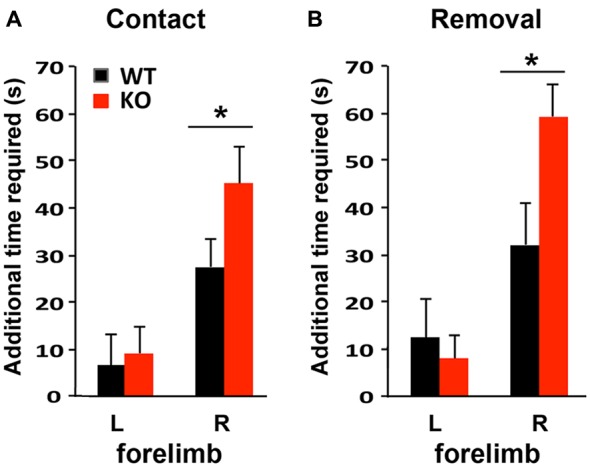
Sensory-motor function is more severely impaired in IRF2BP2KO mice after ischemic injury of the left sensory motor cortex as revealed by the adhesive removal test. Mice were subjected to adhesive removal tests to compare the time needed to remove adhesive tape on the left and right paws 1 week before vs. 4 days after stroke. IRF2BP2KO mice need more time to detect (contact time, **A**) and remove (removal time, **B**) the adhesive tape on their right paw after stroke. *N* = 10–15 mice/group. *p < 0.05.

To compare the functional outcomes after focal ischemic injury of the sensory-motor cortex, mice were subjected to the adhesive removal test 1 week prior to photothrombosis and then again at 4 days after stroke to evaluate sensory-motor functional deficits. Photothrombosis in the left sensory-motor cortex impaired both sensory and motor function of the right forelimb in WT and KO mice, as indicated by the increased time required to detect (contact, i.e., sensory response, Figure 5A) and to remove the adhesive (i.e., motor function, Figure 5B), compared to the contralateral side (left forelimb). Sensory and motor functional deficits were more severe in KO mice.

### IRF2BP2 Is Required for the Anti-Inflammatory Effect of IFNβ

**Figure 6:**
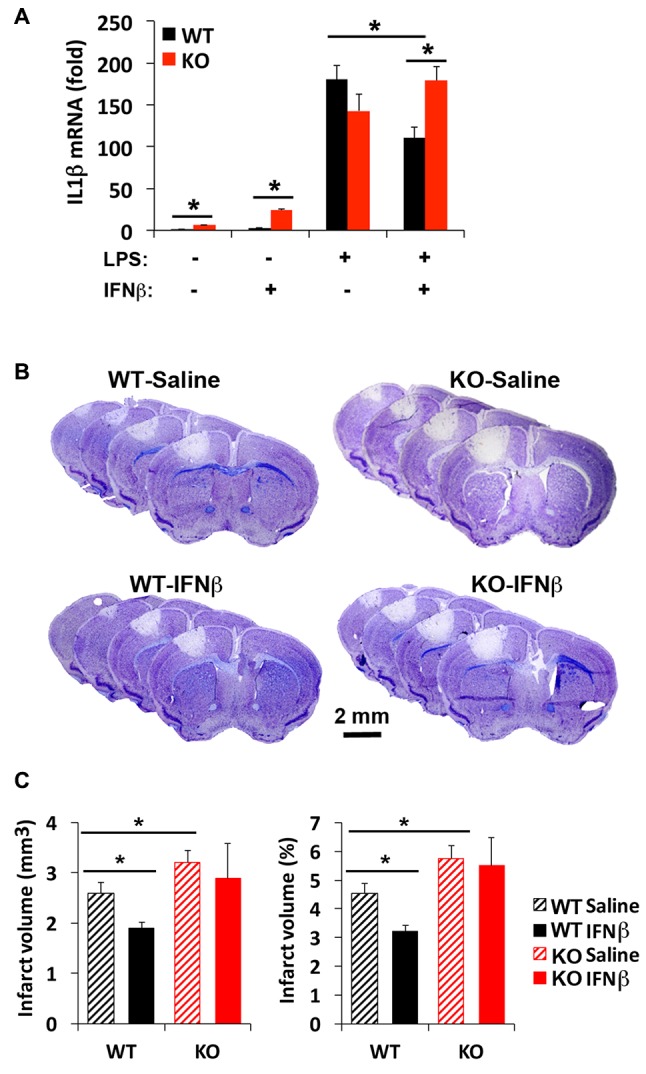
Interferon beta (IFNβ) suppresses inflammatory cytokine activation and reduces stroke injury in WT but not IRF2BP2KO mice. **(A)** Pre-treatment of WT bone-marrow derived macrophages (BMDMs) with IFNβ (100 unit/ml) 2 h prior LPS (10 ng/ml, 4 h) attenuates LPS-induced activation of the inflammatory cytokine IL1β, and this effect is lost in IRF2BP2KO macrophages. IL1β mRNA was normalized to GAPDH. *N* = 4 each. Intravenous delivery of IFNβ (10,000 units) 30 min after photothrombosis reduces lesion volume measured at 4 days after stroke **(B,C)**. This protective effect of IFNβ is absent in IRF2BP2 KO mice. *N* = 10–15 mice/group. *p < 0.05.

Although a beneficial effect of IFNβ to limit stroke injury remains controversial (Maier et al., 2006; Inacio et al., 2015; Kuo et al., 2016), IFNβ is known to limit inflammatory cytokine expression via IRF2 (Hinkerohe et al., 2005; Buttmann et al., 2007). Since there are three corepressors of IRF2, including IRF2BP2, IRF2BP1 and IRF2BPL, it was not clear whether loss of one corepressor IRF2BP2 would affect IFNβ-dependent attenuation of inflammatory cytokine activation. Our prior microarray analysis had shown that IRF2BP2 deficient macrophages express the two other corepressors of IRF2, showing no change in IRF2BP1 but 1.6-fold elevated expression of IRF2BPL (see online supplement Table III in Chen et al., 2015) that could have compensated for loss of IRF2BP2. Thus, we first compared how loss of IRF2BP2 would affect the ability of IFNβ to suppress IL1β expression. Indeed, we found that LPS-mediated induction of IL1β was suppressed by IFNβ in WT BMDM. However, IFNβ failed to suppress the inflammatory response in IRF2BP2 KO macrophages, where an even higher activation of IL1β was observed after LPS stimulation (Figure 6A). Thus, IRF2BP2 is the major repressor of IRF2 to mediate the suppressive effect of IFNβ.

### The Effect of IFNβ to Limit Stroke Injury Requires IRF2BP2

To test whether IFNβ can limit stroke injury following focal ischemia, IFNβ was injected 30 min after photothrombosis and the infarct volume was measured after 4 days. Consistent with the inflammation-damping effect of IFNβ we observed in cultured WT macrophages, infarct volume was reduced by IFNβ in WT mice. However, this protective effect of IFNβ to limit infarction injury was absent in IRF2BP2 KO mice (Figures 6B,C). Thus, IRF2BP2 in microglia/macrophages is necessary to mediate the anti-inflammatory and protective effect of IFNβ on stroke injury.

## Discussion

In the present study, we confirmed that IRF2BP2 limits the inflammatory phenotype in microglia, as we reported previously in macrophages (Chen et al., 2015). IRF2BP2KO mice have delayed regression of infarction and worsened sensory-motor functional deficits, associated with more M1 but fewer M2 microglia recruited to the peri-infarct area. Our study supports the notion that IFNβ administered after focal ischemia limits stroke injury. Importantly, we found that IRF2BP2 is required for IFNβ to attenuate inflammation and to limit ischemic brain injury. These results suggest that optimizing IRF2BP2 levels after stroke, in combination with IFNβ, may be a strategy to promote stroke recovery.

As we reported for IRF2BP2-deficient macrophages (Chen et al., 2015), IRF2BP2-deficient microglia also showed heightened activation of M1 inflammatory markers and reduced activation of many M2 anti-inflammatory markers (Arg1, Fizz1 and YM1). We were surprised to find that IRF2BP2-deficient microglia do not lose their ability to upregulate CD206 in response to IL4, in contrast to IRF2BP2-deficient macrophages (Chen et al., 2015). Thus, CD206 expression is not dependent on IRF2BP2 in microglia; adding to the list of intrinsic differences in gene expression between microglia and macrophages (Hickman et al., 2013). Nonetheless, we observed fewer CD206+ and CD68+ cells after stroke in KO mice. This is likely due to reduced proliferation or recruitment of M2 phagocytic microglia/macrophages and may account for the delay in resorption of the lesion 4 days after photothrombosis, since cultured neonatal IRF2BP2-deficient microglia showed a similar phagocytic activity to WT.

The photothrombosis model of ischemic brain injury replicates several important hallmarks of ischemic stroke in humans. A recent elegant study using histology and MRI in mice clearly demonstrated the presence of a penumbra with brain edema correlated with functional sensory-motor deficits (Li et al., 2014). Although resident microglia, rather than infiltrating macrophages, are the predominant immune cells contributing to microgliosis after photothrombosis (Li et al., 2013, 2014), breaks down the blood-brain-barrier (Krysl et al., 2012) would also permit recruitment of macrophages. Thus, we cannot exclude the contribution of infiltrating M1 (inflamed) IRF2BP2-deficient macrophages to worsen stroke outcomes.

There is a well-documented reorganization of the sensorimotor cortex after photothrombosis (Harrison et al., 2013). This process depends on synaptic pruning by microglia (Paolicelli et al., 2011). The contribution of M2 vs. M1 microglia on synaptic pruning is not known (Hu et al., 2015). Whether absence of IRF2BP2 in microglia that favors M1 polarization impairs synaptic pruning and the reorganization process remains to be determined. Consistent with a previous report (Li et al., 2014), we observed regression of infarction lesion over the course of 4 days in WT mice, associated with recovery of sensorimotor functions. This process was prevented (or delayed) in KO mice. The sustained functional deficits in KO mice at 4 days could simply reflect a persistent larger infarction or an additional defect in synaptic remodeling. Inflammatory cytokines IL1β and TNFα are both significantly elevated in the ipsilateral cortex of KO mice. Pathological levels of IL1β impede synaptic long-term potentiation (Ross et al., 2003) whereas elevated TNFα contributes to early synaptic abnormality in somatosensory cortex in mouse models of experimental autoimmune encephalomyelitis (Yang et al., 2013). Together, these reports confirm a deleterious effect of chronic brain inflammation on brain function. Thus, rapid resolution of inflammation after stroke is desirable for recovery.

IFNβ is known to limit inflammatory cytokine expression and this effect requires the transcription suppressor IRF2 (Hinkerohe et al., 2005; Buttmann et al., 2007). Our study confirmed a beneficial effect of IFNβ to limit inflammation and reduce stroke injury in mice. Importantly, we demonstrate for the first time that the IRF2 corepressor IRF2BP2 is required for IFNβ to attenuate activation of the inflammatory cytokine IL1β. Moreover, other IRF2 corepressors (IRF2BP1 or IRF2BPL) cannot compensate for the lack of IRF2BP2, indicating that IRF2BP2 is the major corepressor for IRF2 to mediate the inflammation suppression effect of IFNβ.

It is important to point out that inflammatory stressors suppress IRF2BP2 expression in macrophages (Chen et al., 2015). Thus, inflammation caused by ischemic brain injury would suppress IRF2BP2 expression and this may compromise the full beneficial effect of IFNβ to limit stroke injury. Recent studies identified several ischemia-induced microRNAs, including miR-107 (Yang et al., 2014; Bhatia et al., 2016) and miR-155 (Arruda et al., 2015), that target and suppress IRF2BP2 expression. Antagonism of miR-155 promotes stroke recovery (Caballero-Garrido et al., 2015), a process that likely involves increasing IRF2BP2 protein levels. Future studies will be required to test whether antagonism of miR-107 and miR-155 can maintain IRF2BP2 expression after stroke to improve the therapeutic effect of IFNβ for stroke recovery.

## Author Contributions

SAC, AH and H-HC designed the experiments. SAC, AH, ZQ, PC and HH performed the experiments. SAC and AH analyzed the data. AFRS generated the IRF2BP2flox transgenic mouse line. H-HC and AFRS wrote the manuscript. DCL edited the manuscript. All authors discussed the results and implications and commented on the manuscript.

## Conflict of Interest Statement

The authors declare that the research was conducted in the absence of any commercial or financial relationships that could be construed as a potential conflict of interest.
